# Contesting Sexual Prejudice to Support Sexual Minorities: Views of Chinese Social Workers

**DOI:** 10.3390/ijerph18063208

**Published:** 2021-03-19

**Authors:** Diana K. Kwok

**Affiliations:** Department of Special Education and Counselling, The Education University of Hong Kong, Hong Kong; dianakwok@eduhk.hk

**Keywords:** social work training, Chinese social workers, sexual prejudice, LGBQ+, heterosexism, social service

## Abstract

Professional development has been recognized as one of the strategies to effectively combat sexual prejudice and negative attitudes against lesbian, gay, bisexual, questioning/queer (LGBQ+) individuals and sexual minorities. Nevertheless, studies related to LGBQ+-inclusive training are rarely found in the Chinese Hong Kong context, where sexual prejudice still prevails without the establishment of antidiscrimination law. Sociocultural considerations, such as religious and parental influences, are obstacles to discussing the reduction of sexual prejudices, both within wider society and social work organizations, without institutional support. This paper aims to understand social workers’ perspectives on prejudice reduction training themes and perceived cultural barriers through qualitative in-depth interviews with 67 social workers. Qualitative thematic analysis yielded the following themes: (1) understanding sexuality; (2) initiating training legitimately; (3) contesting religious and cultural assumptions; (4) resolving value and ethical dilemma; (5) selecting relevant knowledge; (6) implementing diverse training strategies. The study suggests that social workers and service providers need to understand how sexual prejudice is manifested in Hong Kong through unique cultural forces. LGBQ+-inclusive content, addressing updated concepts and prejudice-free language, should be incorporated into the training curriculum. Intergroup contact, professional reflection, and experiential learning are suggested as training strategies (190).

## 1. Introduction

Supporting sexual minorities with professional competence has become an area of concern in social work training. This concern is likely in response to growing attention to health disparities in sexual minority populations [1,2,3,4,5,6]. On the one hand, studies have demonstrated that sexual prejudices and negative attitudes towards sexual minorities among social workers constitute barriers in service provision, inducing possible minority stress and health risks for LGBQ+ clients [3]. On the other hand, it has been revealed that support from social workers and LGBQ+-inclusive policies can enhance mental health and the accessibility of support services [3]. This has given rise to concern over social work training to support LGBQ+ service users better in relation to sexual prejudices [6].

Hong Kong is a city blending Eastern and Western cultures. It has been a special administrative region of the People’s Republic of China since 1997, when China resumed sovereignty at the end of British colonial rule of 156 years. Sociocultural influences, such as Chinese Confucianist values and Western Christianity, are some obstacles to discussing LGBQ+-inclusive rights and the reduction of prejudices [7]. For example, public-funded schools and social work agencies with conservative Christian backgrounds often become sites where the human rights of sexual minorities are denied [8,9,10,11,12,13]. Barrow commented that the Hong Kong government’s progress in enacting legal reforms to protect sexual minorities’ citizen rights has been “seized upon by counter-movements, including religious opposition and parental concern groups” [8] (p. 138).

Sexual prejudices and discrimination happen in “communities, medical and social services, and school locations and is perpetrated by coworkers, family members, religious group members, mental health professionals, and teaching professionals” [10] (p. 447). Given the circumstance, academics and rights-based agencies have called for prejudice reduction policies, tactics, and strategies, including professional development training for social workers to combat sexual bias in social work organizations [5,14]. Among the strategies, training for professional development is identified as an effective means to reduce prejudices against sexual minorities [15,16]. 

Sexual prejudice among social workers have been a long-standing global concern in the last few decades [3,17,18,19], yet it has only recently received attention in Asian cities, like Hong Kong, without addressing social work training themes, especially with a focus on combating sexual prejudice. This study is grounded on concepts of sexual prejudice [20], minority stress [21], and contact theory [22]. Drawing on the findings from qualitative interviews with frontline social workers, the aim of the study is to explore social workers’ perceptions and suggested themes of antisexual prejudice social work training in Hong Kong. 

## 2. Literature Review

### 2.1. Sexual Prejudice and Mental Health of Sexual Minorities 

Social psychologist Herek conceptualized the negative attitudes and sexual stigmas against LGBQ+ individuals as sexual prejudices, which may manifest at both individual and institutional levels. This term “characterize[s] heterosexuals’ negative attitudes toward (a) homosexual behavior, (b) people with a homosexual or bisexual orientation, and (c) communities of gay, lesbian, bisexual people” [20] (p. 1). A “minority stress model” has been emphasized in the extant literature, related to sexual prejudices and the mental health of LGBQ+ individuals [21]. This model suggests that LGBQ+ individuals experience extra stress, in addition to general daily life stress, not because of their sexual identities but because of sexual prejudices and LGBTQ+-hostile societies [21]. Internationally, there are social changes occurring, challenging sexual prejudices, particularly with respect to sexual minorities’ human rights and societal understanding of sexual and gender diversity. The establishment of laws allowing same-sex marriage in more than thirty jurisdictions, including Taiwan in Asia, has reflected such changes [7]. However, as reflected from global and local studies, sexual minorities still experience considerable levels of stigma and barriers. For example, global publications have revealed that sexual prejudices or heterosexist social experiences contribute to minority stress and mental health risks for LGBQ+ individuals, such as sexual health problems, depression, substance abuse, and suicidal vulnerabilities [23,24,25,26,27]. Locally in Hong Kong, Lau and Stotzer found that nearly one-third of the 792 respondents reported sexual orientation-based discrimination, which is associated with negative mental health outcomes [28]. The same research team reported a sample of over six hundred informants facing sexual orientation-based violence two years later [29], with sixty percent reported as victims of nonphysical violence. They also found that the informants’ experiences as violence victims were connected to mental health vulnerabilities. In another study of sexual minority students, the participants suggested that their minority stress and isolation in schools might have been associated with institutional barriers, such as homophobic school cultures with noninclusive school policies [30]. The author suggested that social exclusion of sexual minorities in schools was likely due to sexual prejudices, as manifested through institutional heterosexist cultural and religious values, as well as the absence of professional codes of practice specifically addressing sexual prejudices shown by teaching and mental health practitioners. In light of the mental health risks and minority-group stress of LGBQ+ individuals, as induced by sexual prejudice [21], social work scholars have called for social workers and educators to respond to the needs of sexual minorities [1,6]. 

### 2.2. Sexual Prejudices and Social Workers in Hong Kong

Sexual prejudice among social workers has recently received attention in Chinese societies in Asia [5]. Even though homosexuality has been depathologized for over thirty years and deleted in 2001 from the Chinese Diagnostic Manual of Mental Disorders [10], sexual prejudice against LGBQ+ individuals continues in Hong Kong Chinese society [10,31]. It occurs in the professions of education, health, and social services [10,11]. 

Helping these professionals’ attitudes to sexual minorities have long been under the influence of both Western religious and Chinese Confucian family values [12] Christianity in Hong Kong has had a considerable role in influencing education and social service programs [7,12]. For example, due to Hong Kong’s colonial background, many public-run schools and social service providers are Christianity-associated [7,32]. Administrators in social service agencies have the power to implement or to omit sexual diversity education and service provisions. At the same time, in many Chinese families, same-sex affection is unacceptable as it disrupts the strong Chinese Confucian family value of filial piety (Xiao). Xiao’s principle stresses that Chinese children are responsible for continuing the family line through procreation as a way of showing respect to their parents and ancestors [7]. In regard to legal protection, there are no antidiscrimination ordinances to address the possible sexual prejudices enacted by the general public, social service organizations, and social workers against LGBQ+ service users, even though the social work code of ethics in Hong Kong highlights the importance of the social justice principle [33].

To provide frameworks for social workers, the Hong Kong Social Workers’ Registration Board states that social workers should respect the “dignity of every human being, irrespective of one's…sexual orientation” in its Code of Practice [33] (p. 2). However, there is no mandated training or policy to prevent social workers’ sexual prejudices against LGBQ+ people. Social workers can express their sexual prejudices, both explicitly and covertly, in Hong Kong [5,34]. For instance, one hundred and fifty religious groups, collaborating with over three hundred social workers in Hong Kong, openly express their condemnation of public consultation to endorse an antidiscrimination ordinance that protects LGBQ+ individuals from discrimination [34]. A study of 462 preservice social workers from accredited university social work programs found evidence of prejudices against sexual minorities. The most common factor in predicting differences in these prejudices is religious affiliation. Participants with Christian affiliations reported more unaccepting attitudes than those with no religious beliefs [5]. 

### 2.3. Prejudice Reduction Training for Social Workers 

Sexual minorities are especially vulnerable when laws and policies are not in place to combat sexual prejudices and if health care providers or social workers are not trained properly in sexual diversity issues [5]. Van Den Bergh and Crisp suggested three dimensions of sexual diversity training, namely, attitudes, knowledge, and skills/behaviors, for training competent social workers to work with sexual minorities [35]. Other research indicates that practitioner behavior with sexual minority clients are determined by their knowledge and attitudes. Practitioners who have received professional development training in sexual diversity are more likely to be helpful and to support sexual minority clients [36]. Social work scholars, through their studies, support the notion that a prejudicial attitude can be changed and modified through professional training [4]. A substantial number of studies on training strategies for prejudice reduction are based on contact theory. Allport’s contact hypothesis states that prejudices may be reduced through group interaction with equal group status and common goals [37]. Pettigrew extended the contact theory by suggesting that exposure to educational information and intergroup contacts are both important components of prejudice reduction [38]. Professional training is seemingly one of the many approaches to prejudice reduction, and diverse training strategies based on contact theory seem to be effective in prejudice reduction.

## 3. Research Methodology

### 3.1. Background of Researcher and Participants

Creswell (2007) suggests that a qualitative approach should be chosen when a researcher plans to study silenced voices from a group, such as sexual minorities and issues related to sexual prejudices within social services [39]. The researcher is a female, registered Chinese social worker in Hong Kong, an ally to the Creswell approach. The transcripts for this paper were collected while she was conducting a larger research project on understanding sexual prejudice in Hong Kong, with the aim to explore both social workers’ and sexual minorities’ experiences related to sexual prejudice in social services. The researcher is also working collaboratively with community organizations to provide sexual and gender diversity public education. Her interest in this topic stems from a concern to improve social work practice with sexual minorities in Hong Kong. This paper focuses on the transcripts from 67 Chinese social workers in Hong Kong, with the aim of exploring social workers’ perspectives on prejudice reduction training themes and perceived cultural barriers with the following research questions: (1) What are social workers’ perceptions or understanding of sexuality knowledge? (2) What are their experiences with cultural forces when encountering sexual prejudices in the social work context? (3) What are the suggested contents and themes relating to sexual prejudice reduction for social workers? To answer the specific research questions of this paper, the “qualitative description approach” was adopted. This approach has been applied in social work and health studies [40,41] (p. 867). It is “ideal for effectively identifying observations and constructs emerging from narrative-based data” and is not aiming at “a specifically defined lens or theoretical approach to data interpretation” [40] (p. 867). In line with this approach, purposive sampling was adopted, with “a broad range of phenomenally and/or demographically varied cases”. The sample size within this approach varied based on the research questions, ranging from 24 [42] and 60 [40] to 108 informants [43].

Regarding this study, most of the informants were female (68.7%), with the rest (31.3%) being male. The informants’ ages ranged from 21 to 45 years, with a mean age of 30.6 years. The participants were recruited through purposive sampling in several ways to obtain information-rich cases [44], for instance, in-service social worker training programs in nongovernment organizations (NGOs), different settings of social services, such as school and college counseling and social work teams, youth services, sexual-health- and diversity-related services, rehabilitation and medical services, family service, and residential, outreach, and crisis services. The participants came from diverse settings, including youth, family, sexual health, medical, diversity/ethnic minority, crisis-support services (Table 1).

### 3.2. In-Depth Interview and Data Collection

Ethical clearance was completed before data collection. Informed consent and confidentiality principles were explained to the informants. A semistructured interview guide was planned, with key domains connecting to social worker perception of sexuality knowledge, perceived examples of sexual prejudices observed or experienced in social service contexts, social work training contents, strategies, and barriers. Amendments of interview questions were made after seeking feedback from LGBQ+ members and social workers. The process of the interviews was collaborative, supported by a semistructured interview guide. Informants were able to direct the focus of the interview at the beginning with open-ended questions. Examples of these open-ended questions included “please share with me as much detail as possible your perceptions on sexuality-related concepts” and “what are the barriers in providing sexual diversity education for social workers in Hong Kong”. If informants felt anxious in responding to open-ended questions, more structured questions would be used to lessen their anxious feelings, such as “based on some of your work-related experiences with LGBQ+ service users, what do you think would be the most useful content and strategies to be included in a social work training workshop on reducing sexual prejudice”? As all the informants were Chinese and spoke Cantonese, the interviews were carried out in Cantonese and tape-recorded. All the tapes were transliterated in Chinese by a research assistant trained in qualitative research. Narrative transcription was done in Chinese, with identifying data deleted from the transcriptions. The data collection was initially planned with small groups, but as the study progressed, some informants favored individual interviews as this method helped them to feel safe, protected their privacy, and enabled them to feel more comfortable discussing sexual prejudice issues within the social work context. 

### 3.3. Data Analysis

The data were analyzed using qualitative thematic analysis, which has been described as “a method for identifying, analyzing and reporting patterns (themes) within data” [45] (p. 78). The first step involved the researcher and a research assistant reading the interview transcripts to get a general impression of the informants’ experiences. At the second step, the researcher and research assistant discussed the initial coding frame to reach an agreement on the coding frame. The initial coding frame included the following major items: (1) sexuality knowledge and concepts; (2) experiences and observations of sexual prejudice examples within social services; (3) experiences and perceptions with sexual diversity training, including curricula and strategies; (4) sociocultural barriers in conducting sexual diversity training. At the third stage, the coding frame was input into NVivo (QSR International, Doncaster, Australia), a software for analyzing qualitative data. Based on the coding frame, the data were coded and analyzed through identifying and extracting themes within the interview transcripts related to the understanding of sexuality knowledge, cultural forces related to sexual prejudice, and social work practices, such as facing value dilemmas and training issues within the social service context. The fourth stage involved reviewing extracted themes with the coded data. The coded themes were then reviewed again and again in meetings to reconsider if the themes were agreed upon between the researcher and the research assistant [44,45]. Methods employed to safeguard the rigor of this research were the following: (1) the transcripts were checked with the informants after each interview to see if the information collected reflected their experiences; (2) the researcher obtained rich descriptions of the informants’ responses through in-depth interviews; (3) prolonged engagement and observations in the LGBQ+ and social work communities [44,46] through ethnographic participation in LGBQ+ youth mutual help programs and collaboration with LGBQ+ groups in providing sexual and gender diversity workshops for parents, educators, and social workers.

## 4. Findings

**Figure 1:**
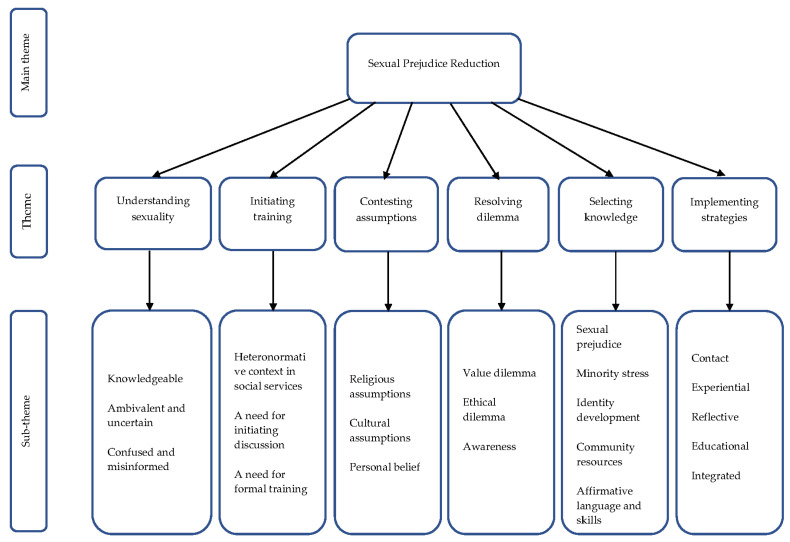
Quotes from informants’ transcripts.

This study aims to understand social workers’ perspectives on prejudice reduction training themes and perceived cultural barriers. The resulted themes included (1) understanding sexuality; (2) initiating training legitimately; (3) contesting religious and cultural assumptions; (4) resolving value and ethical dilemmas; (5) selecting relevant knowledge; (6) implementing diverse training strategies. Quotes from the informants’ transcripts will be used to illustrate the themes (Figure 1).

### 4.1. Understanding Sexuality 

Informants with diverse backgrounds and understanding of sex, gender, and sexuality came to the interview. The ones who were considered knowledgeable on the subjects typically had community connections and were relatively open and comfortable in sharing their opinions. Those who were less knowledgeable usually showed signs of misconceptions, with ambiguity and unsureness in expressions. 

The ones with direct service experience had a broad understanding of sex, gender, and sexuality. Besides working with gays, lesbians, and bisexuals, some had dealt with intersex and transgender groups. They defined sex and gender beyond binary resolution. They were aware that society’s prejudiced views, such as sexualizing LGBQ people, can cause them to be denied service accessibility and induce mental health stress for them.

*“I am very ready to discuss…I learnt that sex, sexual orientation, and gender can go beyond binary definitions… Sexual activities and pleasure are part of the well-being and life experience of sexual minorities too… Hong Kong society is still oppressive; there are distorted views and concepts of sex and sexuality, such as linking gay men to sex addiction, illness, or sin… Misconceptions about intersex people and transgender populations… Despite more open discussions, it is still generally unsafe for sexual minorities to come out of the “closet”… In social work, there is a service gap, but many social service agencies are not aware of that… neglecting sexual minorities’ service accessibility and mental health concerns.” (Female social worker from a sexual health service)*

*“Sexuality is fluid. Your sexuality status at birth can change at a later stage in life…I understand that some social workers may apply heterosexual frameworks to intimate relationships of same-sex couples, but this is inappropriate…Sexual orientation is a holistic concept, with dimensions of emotional and physical attractions, as well as identity issues.” (Male social worker from a youth service)*

There were informants who felt ambivalent when discussing concepts related to sexual and gender diversity. They were ready to support sexual minorities yet did not have relevant knowledge. For example, some felt unsure and unprepared to understand a client’s stress, having no concepts of sex, sexual orientation, gender expression, or identity. They might, for example, misunderstand a trans man as a lesbian with masculine gender expression.

*“I was confused…I had a female client who had a masculine appearance…I thought she was a lesbian…She was checked into a substance abuse residential service. This client felt very stressed and uncomfortable to be placed in a room with female roommates...She once shared that she was a man… I do not have relevant knowledge to understand the situation.” (Female social worker from a crisis care service)*

*“We have no relevant training. I worry that I cannot fulfill my role…Even though we are eager to help all clients, gays and lesbians, we have no understanding about them. We have no relevant knowledge, skills, or access to resources. We want to, but we do not know how to help.” (Male social worker from a family service)*

A very small group of informants inaccurately confused the concepts of sexuality and intimacy. They also considered sexual orientation change therapy as one of the treatment approaches, with no education about the possible mental health harm that this kind of intervention can bring to sexual minorities.

*“If you are gay and are attracted to a person, you can have emotional intimacy with that person. But if the intimacy leads to sex, it would be unacceptable for me... you need to consider the impacts of such sexual behavior on society...gay sex will spread STDs.” (Female social worker from a family service)*

*“I believe it is the client’s choice if he (or she) wishes to change his (or her) sexual orientation… it is a feasible method of treatment or intervention.” (Female social worker from a school service)*

### 4.2. Initiating Training Legitimately

Most informants recognized that sexual prejudices against sexual minorities exist in the current social services, with the heterosexual perspective being the norm. This is manifested in the design of intake forms, in ways of engaging clients, in facilitating access to services, and the like. They found that most training programs for social workers are designed under heterosexist lenses without considering LGBQ+ issues. The informants expressed their need for professional training in LGBQ+ issues, with the formal and official support of the Hong Kong Government’s Social Welfare Department.

*“Social work colleagues with low levels of awareness may make improper comments during the engagement stage of the helping process with sexual minority clients. For example, comments regarding a social worker’s support of sexual orientation change therapy…The client–social worker relationship is a reciprocal one, and the social worker’s awareness of sexual minority issues will have a strong influence on the sexual minority clients’ trust and willingness to disclose their concerns.” (Male social worker from a youth service)*

*“I observe that recent youth work training mostly focuses on general mental health concerns, such as depression. There is minimal, if any, discussion of socially-based stress for gay and lesbian clients…It is inevitable that the service provisions and training themes are affected by agencies’ religious, cultural, and family values when it comes to sexuality issues…even if some social workers want to support sexual minority service users in their outreaching work, they could only do it unofficially in order to protect themselves from the agency’s scrutiny …It appears to me that sexual diversity training should address religious and agency barriers in addition to the foundation concepts of sex and sexuality.” (Female social worker from a youth service)*

*“It is noteworthy that some social welfare agencies worry about their sources of funding as well as their service users making complaints, and so they regard sex-related issues as taboo; this can hinder social workers' attempts to offer services to help sexual minorities or for agencies to initiate training for their staff.” (Female social work from a crisis service)*

*“Social welfare agencies and the Hong Kong Social Workers’ Registration Board provide no guidelines for preventing homophobia or heterosexism among social workers. If the government is not taking the lead on this issue, service agencies will not change the existing norm of practice or allocate resources for training to do so…From my experience, I can say that sexual prejudices against sexual minorities do exist in different professions working in multidisciplinary teams, such as medical and allied health teams.” (Male social worker from a medical social service)*

### 4.3. Contesting Religious and Cultural Assumptions 

Some informants have witnessed prejudicial comments against sexual minorities in their service or training settings. They thought that some social work colleagues or educators tried to justify their sexual prejudices against sexual minorities with religious values and cultural reasons. One interviewee reported that since 90 percent of social workers in a particular service unit had religious beliefs, religious elements were added to meetings, supervision, and daily services. Colleagues who did not have religious beliefs felt they were being forced to comply. The informants, therefore, promote the need to develop awareness and to have greater sensitivity to personal beliefs about sexuality in social work professional practice and training, particularly in contesting and re-examining cultural and religious assumptions about sexuality.

*“Sexual prejudice against sexual minorities was often justified by some social workers’ cultural beliefs and social norms. The value of filial piety is strong in Confucian culture, with the belief that one has the duty and responsibility to carry on the family name... it is also related to our practice of emphasizing collectivism, not individualism... expecting everyone to have the same way of thinking...Chinese culture is about harmony, and one should not deviate from the majority.” (Male social worker from a sexual health service)*

*“The professor, also a registered social worker, openly stated the Catholic position in my social work ethics class, perceiving nonheterosexuality as an immoral sexual act, which violated the Catholic protocol of moral standards…There were gay students in the class, who might have felt very uncomfortable and oppressed…I challenged this point of view…When a social work professor or a social worker’s values are in conflict with the professional values, other than having a safe space for discussion, we need to contest the assumptions.” (Female social worker from a sexual health service)*

### 4.4. Resolving Value and Ethical Dilemma

The informants suggested that it is important to work on attitudes and awareness through revisiting codes of practice of social work and identifying value and ethical dilemmas. Equally important, they suggested the need to develop an understanding of how social–cultural factors in Hong Kong contribute to social workers’ attitudes to and understanding of sexuality and how these social–cultural forces affect the life experiences of sexual minorities.

*“A social worker may be brought up in a traditional way or he has a strong religious faith based on school education or family/church influences. Such factors may override the code of practice and lead to prejudice against sexual minorities…Continuous awareness training is important…(social workers) lacking the awareness of their influences will not understand how the work on sexual minority clients can be affected, nor are they prepared to handle challenges to their own values. These can easily lead to discriminatory behaviors that will hurt the clients…Training may include personal awareness exercises, frontline case discussions to identify value or ethical conflicts.” (Male social worker from a school service)*

### 4.5. Selecting Relevant Knowledge

The informants revealed that foundation knowledge about sexuality, e.g., definitions of sexuality, sex, and gender, should be included. Additionally, contemporary sexuality perspectives and updated research studies should be introduced. Other topics, such as concepts relating to prejudice, minority stress, sexual/gender identity development, and community resources, should be made known to social workers. Affirmative language and skills for interacting with sexual minority students should be informed by current developments reported in the literature.

*“All social work employees ought to be given relevant training; this is essential. That is before you (the agency/NGO) can claim to be LGBT-friendly, at least one to two colleagues of every center of the entire social service agency have to go through relevant sexual diversity training…including how to deal with societal prejudice, new knowledge on demedicalization and depathologization of sexuality in mental health literature.” (Female social worker from a crisis service)*

*“Multidimensional perspectives should be explored during the training process. When we actually work or meet with the service clients, we should automatically take on a multidimensional perspective rather than a single perspective to analyze the issue…definitions and concepts, contemporary sexuality perspectives, and updated research studies should be involved…Probably some inclusive languages or cultural terms.” (Female social worker from a school service)*

### 4.6. Implementing Diverse Training Strategies 

The informants suggested the use of contact, experiential, reflective, and educational strategies. Regarding the contact approach, role model narratives, videos, community outreach, and panel guest speakers on diverse life experiences of sexual minorities in the social–cultural context of Hong Kong Chinese society would be helpful to them. Reflective and experiential discussions about practice/values/ethical dilemmas and media constructions of sexuality are all potential strategies for exploring ways to resolve value discrepancies and ethical dilemmas. Likewise, the informants recommended that educational materials, such as research findings, theories, and models, are equally essential to equip them with the foundation and updated knowledge.

*“I prefer a mixed approach to training strategies. Use of information can include facts and myths, from updated sexuality research in international and Chinese contexts, and interactive question-and-answer formats. The acronym of LGBTQIA+, sexual and gender fluidity, can be illustrated through role models in case studies, with narratives and videos…When training is provided, we shall give examples of how to uphold social work professionalism not masked by one’s personal values.” (Male social worker from a rehabilitation service)*

*“Community visits or panel speakers could be helpful…these could be used to promote an understanding of sexual minority cultures and resources. In my experience, experiential games, role plays, or use of online platforms could be used to enhance engagement.” (Male social worker from a family service)*

*“Critical reflection is needed to challenge the existing model of social work intervention, especially when the issues around sexuality, sexual behaviors, and gender are discussed; this needs a safe and encouraging atmosphere… holding reflective discussions on ethical dilemmas between values and practices could be potential strategies to address the gaps in knowledge and understanding.” (Female social worker from an ethnic diversity service)*

## 5. Discussion

We are aware of the study limitations. The participants and informants were specifically selected through purposive sampling. Consequently, it is likely that the participating social workers were more ready to talk and might be more tolerant and inclusive than other social workers about understanding and supporting sexual minorities. In addition, it is possible that institutional and cultural constrictions might have inhibited participating social workers from conveying their experiences fully in the study. This article contributes to knowledge advancement by adding to the literature on antisexual prejudice social work training in sexual diversity studies, especially prejudices targeting LGBQ+ individuals in social work contexts in Hong Kong Chinese society. 

This study has revealed themes similar to those identified in European cities and North American countries regarding societal attitudes towards LGBQ+ individuals. Homophobic and prejudicial attitudes have been found among social service professionals and the general public [1,47]. Moreover, the manifestation of sexual prejudices may also lie within institutions; it has been documented that those in social service programs, social work teaching, and service delivery systems often overlook LGBQ+ individuals’ needs [3,47,48]. There is evidence not only that sexual prejudices against LGBTQ+ individuals exist and are prevalent but that, in line with the minority stress model, these prejudices can have an impact on mental health [21,26]. This has certainly been found to be the case for Chinese LGBQ+ individuals in Hong Kong, for whom facing multiple cultural barriers may internalize the heterosexist message, which, in turn, will affect their mental health [10,30]. 

Despite the fact that comparable themes of intolerance and sexual prejudice against LGBTQ+ people have been found in other cultures, the circumstances seem to be harder for Chinese LGBTQs in Hong Kong than for their counterparts in places with supportive programs to back them up in contravening homophobia, heterosexism, sexual prejudice, and minority stress, such as the GSAs (gay–straight alliances in the USA), overt ethical guidelines for social workers to contest homophobia, and training to address sexual and gender diversity in service users. The ethical guidelines mentioned in these studies, for example, the Code of Ethics from the National Association of Social Workers, states explicitly that "social workers should not practice, condone, facilitate, or collaborate with any form of discrimination on the basis of [...] sexual orientation" [49] (sec. 4.02). However, the heterosexist social service system and the institutional heteronormativity in wider Hong Kong society add extra difficulty to establishing similar support projects or explicit codes of ethics to prevent sexual prejudice in social workers and service providers. Consequently, many social workers feel unsafe discussing sexual diversity issues or providing explicit information on sexuality to students and parents due to opposition from principals, parents, or agency boards of directors with religious connections. The lack of explicit codes of practice and ethics relating to sexual prejudices manifested by social workers and social service agencies can make it more difficult for social service professionals to support LGBTQ+ service users to transgress prejudices, mental health vulnerabilities, and the stress associated with being in the minority [5,10]. 

Since we can find sexual diversity models and programs of social work training in sexual diversity at the international level and in other countries that are over two decades old [4,35], social work informants in current Hong Kong research can be offered further understanding and insights on how sexual prejudice is presented through sociocultural influences within the social service settings in another cultural context. The informants revealed a need to support social workers in re-examining dilemmas, ethics, and value issues related to cultural and religious influences. 

To help train competent social workers with sexual diversity knowledge and skills, the informants recommend that the Hong Kong Government’s Social Welfare Department should take the initiative in leading the training. Finally, diverse strategies, including outreach and contacts, experiential reflections, and education, rather than a single educational approach, are proposed as key strategies to enrich the participants’ training experiences.

## 6. Recommendations

### 6.1. Starting with Official Guidelines

Sexual minorities are not protected equally, as their heterosexual counterparts are, within the Equal Opportunities Ordinances. There has been no antidiscriminative legislation targeting homophobic discriminations or sexual prejudice in Hong Kong [10]. Equally missing is a code of ethics for social workers and social service organizations on homophobic and prejudiced practice in social services [5]. Without legal protection and an explicit code of ethics to prevent sexual prejudices in social service providers and practitioners, LGBQ+ individuals continue to encounter barriers to receiving supportive mental health and social services. Likewise, it is suggested that policies with equal opportunity principles to address sexual prejudice in social service settings through antisexual prejudice campaigns should be initiated by the Social Welfare Department or the Hong Kong Council of Social Service; a zero-tolerance policy on sexual prejudice is recommended. Social service agencies could establish explicit guidelines for social workers, making it clear that both covert and overt sexual prejudices, such as heteronormative assumptions about sexual orientation or direct verbal homophobic bullying, are not tolerable and, in fact, can be traumatically harmful to LGBQ+ individuals. 

### 6.2. Implementing Sexual Diversity Themes in Training

In Hong Kong, the values of social justice and respect for diversity are required topics for social work curricula [33]. It may be valuable for the Social Welfare Department to encourage social service administrators to support social workers participating in training workshops organized by the Lady Trench Training Institute of the Social Welfare Department or to support individual agencies in developing such workshops for their social work employees within professional development contexts, using the social work training funds given by the government. In developing their professional value systems and delivering quality training programs, it is recommended that educators of professional training programs incorporate LGBQ+-inclusive content to nurture social workers, with a safe space to talk about dilemmas related to values or ethics. It is equally important to conduct awareness training for social workers to be aware of how familial, cultural, and religious values impact their perceptions on sexual diversity issues. It may be useful to involve content and strategies that consider updated sexuality concepts and research. It may be beneficial for training curricula on sexual diversity to include content with advocacy work, in addition to a focus on individual mental health [50]. In terms of advocacy to challenge institutional sexual prejudice, scholars suggest the discussion of a change of oppressive practices implanted in the social service, education, and legal systems that discriminate against LGBQ+ individuals [50]. Diverse training strategies, such as outreach, experiential reflections, and intergroup contact, can be explored. These suggestions are also included in previous sexual diversity training programs, telling us that self-awareness, reflective discussion, education, and contact and outreach to sexually expansive communities are central elements for enhancing professional competence [15,51].

## 7. Conclusions

Despite the study’s limitations, it is the first in an East Asian context and in Hong Kong to explore the perspectives and experiences of social workers regarding the content of sexual diversity training or education to address sexual prejudices and to support sexual minorities within social services. The research results add to the growing body of work on sexual diversity education in social work practice, aiming to reduce sexual prejudices. There is a compelling need for NGOs, the Council of Social Services, and the Hong Kong Government to initiating mandatory social work training to support LGBQ+ individuals against sexual prejudice. It is recommended that they take leadership roles in this. The discussion in this article may raise the awareness of social work educators, social workers, and broader Chinese society to attend to the sociocultural impacts of Chinese people’s sexuality. To realize the widespread sexual prejudice within LGBQ+ individuals’ cultural context would enhance social workers’ sensitivity and facilitate them in developing competence to deliver prejudice-free and inclusive services.

## Figures and Tables

**Table 1:** Informant demographics (N = 67).

Characteristic	%	(*n*)
Gender		
Male	31.3	(21)
Female	68.7	(46)
Sexual orientation		
Heterosexual	77.6	(52)
Homosexual	11.9	(8)
Bisexual	4.5	(3)
Age group		
21–25	26.9	(18)
26–35	49.3	(33)
36–45	22.4	(15)
Religious affiliation		
No religious affiliation	47.8	(32)
Christian	41.8	(28)
Catholic	6.0	(4)
Buddhist	1.5	(1)
Muslim	1.5	(1)
Service group		
Children and youth service	23.8	(16)
School and college service	22.4	(15)
Family and residential service	15	(10)
Sexual-health- and diversity-related service	15	(10)
Rehabilitation and medical service Outreach and crisis service	11.9 11.9	(8) (8)

*Note.* Percentages may not add up to 100% because not every informant provided answers to each question and because of rounding.
